# Molecular‐Level Pore Engineering for Precise Separation via Active Solvent‐Mediated Interfacial Polymerization

**DOI:** 10.1002/advs.77014

**Published:** 2026-08-05

**Authors:** Feng Li, R. M. G. Rajapakse, Yuxin Mou, Deyin Hou, Min Yang

**Affiliations:** ^1^ National Engineering Research Center of Industrial Wastewater Detoxication and Resource Recovery Research Center for Eco‐Environmental Sciences Chinese Academy of Sciences Beijing People's Republic of China; ^2^ University of Chinese Academy of Sciences Beijing People's Republic of China; ^3^ Department of Chemistry University of Peradeniya Peradeniya Sri Lanka

**Keywords:** interfacial polymerization, microemulsion, nanofiltration, solvent effect

## Abstract

Polymer membranes typically face various selectivity demands in practical separations. Tuning the selectivity largely relies on regulating the pore size and distribution in membranes without disrupting the integrity of the polymer network. While monomer design or additive incorporation shows advances in pore tuning, homogeneous sub‐nanometer pore tuning in polymer matrix remains challenging as traditional solvents act solely as diffusive media, leading to uncontrolled polymerization. Here, we report an active solvent‐mediated interfacial polymerization strategy that enables molecular‐level pore engineering of membrane selective layer. By utilizing the microemulsion, a special solvent, we show that ∼1 nm microemulsions induces localized quasi‐homogeneous polymerization, templating molecular‐level pores within the polyamide network. The resulting membranes exhibit excellent water permeance of 58.7 L m^−2^ h^−1^ bar^−1^ with NaCl‐Na_2_SO_4_ selectivity vs. water permeance surpasses the current upper bond. The strategy also works for hollow fiber membranes which show no performance decline even scaling up to 1‐inch modules. Positron annihilation spectroscopy, small‐angle x‐ray scatterings, molecular dynamics, CFD and other experimental results reveal that patterned surface, enhanced free volume and narrower pore size contribute to the enhanced performance. This active solvent‐mediated strategy provides a versatile and scalable method for enhancing membrane performance across water treatment and chemical separations.

## Graphical abstract

We demonstrate that microemulsion solvents can actively template molecular‐level pores within selective layer during interfacial polymerization. By creating localized quasi‐homogeneous polymerization zones, the membrane achieved a 7.1‐fold enhancement in water permeance with increased selectivity. This breakthrough enables precise selectivity tuning and offering a scalable pathway to the development of next‐generation separation membranes.

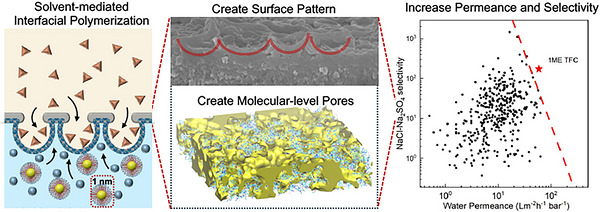

## 1 Introduction

Polymeric pressure driven membranes are essential in resource recovery during wastewater treatment processes, especially for high salinity industrial wastewater, which offer advanced solutions for global water scarcity challenges [1, 2]. Nanofiltration (NF) membranes hold a key position between reverse osmosis and ultrafiltration membranes, making them particularly valuable for removal of divalent ions, small organic molecules, and partial desalination while operating at moderate pressures [3, 4, 5, 6]. The performance of these membranes is governed by the dense, crosslinked selective layer, which in thin‐film composite (TFC) membranes is typically formed continuously through interfacial polymerization (IP) between an amine monomer in an aqueous phase and an acyl chloride in an organic phase [7, 8, 9]. While conventional IP has been widely successful, it offers limited control over the membrane microstructure with board pore size distribution and random free volume [10, 11, 12]. This structural heterogeneity creates a trade‐off between permeability and selectivity, where higher water permeability usually comes at the cost of reduced solutes rejection [13, 14, 15]. Overcoming this trade‐off requires precise structural control of the membrane pore architecture at the molecular level [16, 17, 18, 19].

The key challenge is how to regulate the reaction environment to generate molecular‐level pore sizes in membranes without disrupting the continuity of the cross‐linked network of polyamide [20, 21]. While recent advances have focused on monomer design or additive incorporation [20, 22, 23], the solvent phase (usually water) in conventional interfacial polymerization primarily serves as a passive carrier, creating a sharp interface but not actively participating in structure formation. However, solvent can influence reaction pathways according to recent advances in understanding solvent effects at interfaces [24, 25]. It can modify the free energies of reactive species via solvation, directly participating as co‐catalysts in bond formation, and competitively interacting with active sites. Utilizing these solvent effects offers an underexplored opportunity to control membrane microstructure during interfacial polymerization [26, 27].

Microemulsions represent thermodynamically stable, isotropic dispersions of immiscible liquids with molecular scale droplets (∼1 nm) [28, 29], offering unique advantages as both solvent and soft templates in interfacial polymerization. Unlike conventional solvents, microemulsions act as solubilizers that create organized molecular assemblies accommodating both hydrophobic and hydrophilic components simultaneously, thereby accelerating monomer diffusion and reaction kinetics at the water‐organic interface [30, 31]. This enhanced solubilization capability significantly increases the local concentration of reactive monomers at the polymerization interface, while the thermodynamic stability of microemulsions ensures consistent templating throughout the IP process. Recent studies have explored various emulsion systems in IP, including oil‐in‐water emulsions containing organic solvents [32, 33], which have demonstrated improved membrane permeability and altered surface morphology. However, these conventional emulsion systems suffer from thermodynamic instability and large droplet size. Most importantly, they only act as additives rather than solvent itself. The fundamental difference lies in how the monomer interacts with its surrounding medium. In conventional emulsion‐assisted IP, the aqueous phase remains bulk water, which provides an unmodified aqueous environment regardless of what emulsion droplets are dispersed within it. In contrast, when a microemulsion replaces the entire aqueous phase, the monomer's solvation shell and diffusion kinetics are directly altered at the molecular level. This creates an unprecedented dual regulation of monomer supply. This regulation of monomer diffusion from both sides of the reaction interface represents a fundamentally different polymerization mechanism rather than an additive modification.

In this work, we demonstrate an active solvent‐mediated interfacial polymerization strategy that utilize solvent effects to overcome the permeability‐selectivity trade‐off in polyamide nanofiltration membranes. By using thermodynamically stable 1 nm microemulsions as the solvent phase during IP process, we create organized solvent domains, utilizing these domains to induce localized quasi‐homogeneous polymerization, and trapping these molecular‐scale cavities within the polyamide network. Through comprehensive characterization including scanning electron microscopy, atomic force microscopy, positron annihilation spectroscopy, and small‐angle x‐ray scattering, we show how these solvent‐mediated effects translate into enhanced membrane performance. The optimized active solvent‐mediated membranes achieve up to 7.1‐fold enhancement in water permeance (58.7 L m^−^^2^ h^−^^1^ bar^−^^1^) while modifying the selectivity. Furthermore, we validate the practical use of these membranes through selective separation of valuable components in simulated industrial wastewater. This approach provides molecular‐level control over membrane pore structure with enhanced free volume and interconnected transport pathways while modifying the size‐sieving capability required for precise separations.

## 2 Results and Discussion

**FIGURE 1:**
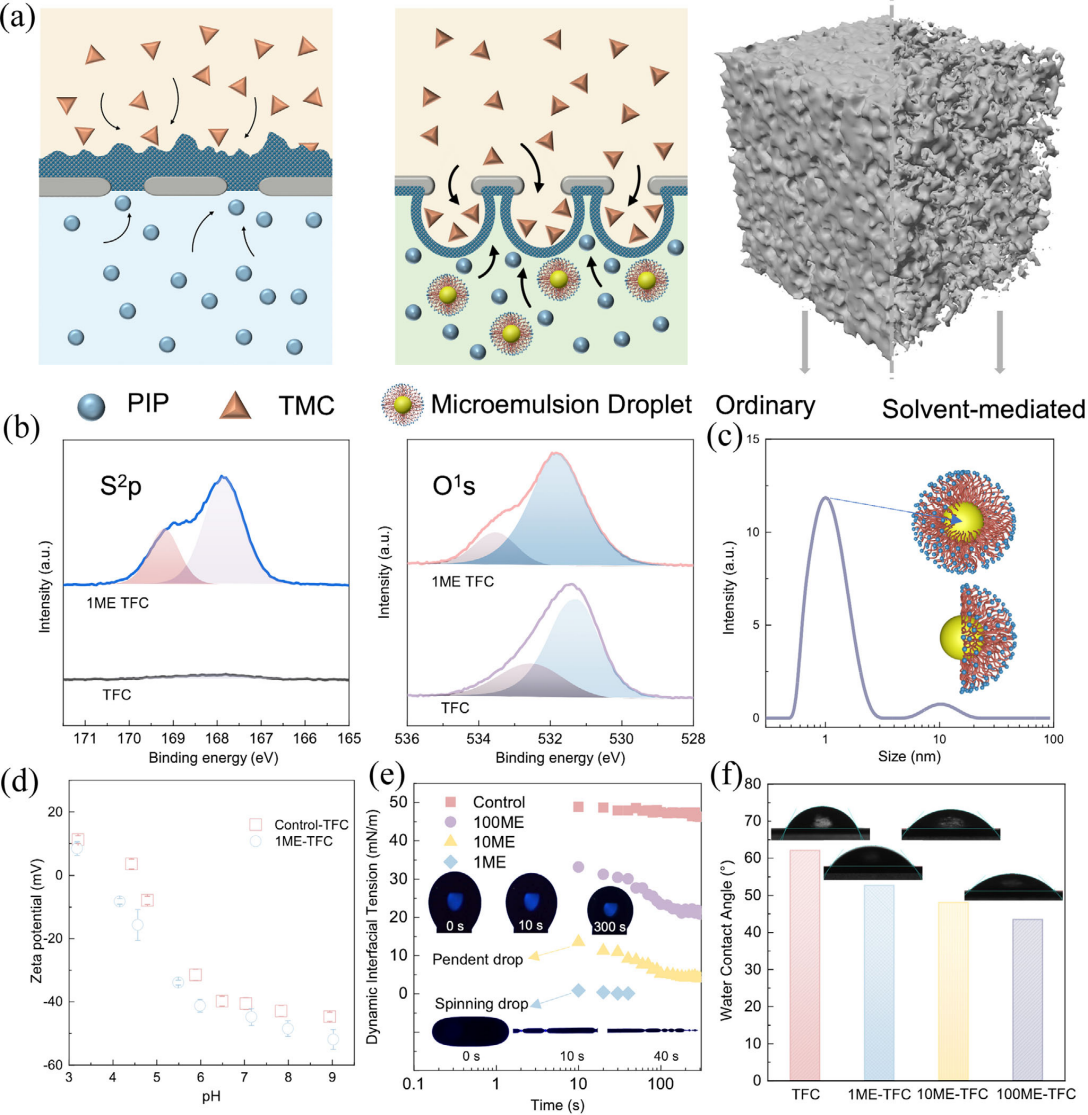
Active solvent‐mediated interfacial polymerization and membrane surface chemistry. (a) Schematic comparison of conventional interfacial polymerization (left) showing typical rough interface formation between aqueous monomers (blue spheres) and organic‐phase TMC (orange triangles); active solvent‐mediated system (middle) demonstrating the formation of bowl‐shaped region at the reaction interface with concentrated monomer regions; and the resulting porous membrane selective layer structure (right) exhibiting enhanced porosity with interconnected channels. (b) S^2^p (left) and O^1^s (right) XPS spectra of control‐TFC and active solvent‐mediated (1ME‐TFC) membranes. (c) Dynamic light scattering based size distribution of 1ME microemulsions. (d) Zeta potential measurements as a function of pH for control‐TFC and 1ME‐TFC membranes. (e) Dynamic interfacial tension measurements. Pendant drop tensiometer was used for control, 10ME and 100ME system, with representative drop images demonstrating progressive shape deformation with decreasing interfacial tension. Spinning drop tensiometer was used for 1ME system where ultralow tensions (<1 mN/m) prevented stable pendant drop formation. The inner representative drop images show that the drop gradually dissolves in the microemulsion solvent. It should be noted that 1ME represents the microemulsion with droplet size ∼1 nm, 10ME represents nanoemulsion with droplet size ∼10 nm, 100ME represents coarse emulsion with droplet size >100 nm. (f) Water contact angle measurements showing improved hydrophilicity with increasing microemulsion concentration.

An oil‐in‐water microemulsion system (SDS/1‐butanol/n‐hexane/water) was used to create stable, monodisperse droplets of approximately 1 nm. In our active solvent‐mediated interfacial polymerization strategy, these microemulsions replace the conventional aqueous phase. We hypothesized that the microemulsion would assemble at the oil‐water interface, physically deforming the reaction zone and creating bowl‐shaped (as evidenced by Figure S1a), localized quasi‐homogeneous polymerization sites (Figure 1a). Then polymerization occurs around these spherical templates. At the same time, microemulsion‐mediated cavities were trapped in the polymer matrix. As visualized in the 3D illustration (Figure 1a, right), these effects result in a highly reticular polyamide architecture.

The incorporation of microemulsion into the polyamide network was confirmed by x‐ray photoelectron spectroscopy (Figure 1b). The S^2^p spectrum clearly demonstrates the existence of sulfur in the selective layer, which is due to the trapped microemulsion containing sodium dodecyl sulfate (SDS). The 1ME‐TFC membrane shows a distinct S^2^p signal with two distinct peaks at approximately 168–169 eV. These binding energies are characteristic of sulfonate groups (─SO^3−^) from SDS. Furthermore, the O1s spectra revealed changed chemical environments, with a slightly different peak profile suggesting that the organic components of the microemulsion actively engaged in the local solvation of reactive species. This modified solvation environment potentially lowers the activation energy for the amine‐acyl chloride reaction by providing a less polar reaction domain [34, 35]. The presence of this additional spectral component in the 1ME‐TFC sample supports the incorporation of microemulsion and suggests changes in the local chemical environment of the polyamide network. Dynamic light scattering further confirmed that the microemulsion droplets exhibited a narrow size distribution centered at approximately 1 nm (Figure 1c), verifying their monodisperse nature and suitability as molecular‐scale templating agents. To directly probe how the microemulsion alters monomer behavior, we performed Raman spectroscopy (Figure S8) and UV–vis absorption measurements (Figure S9). Raman spectroscopy reveals molecular‐level solvation differences between the microemulsion and conventional aqueous environments. PIP dissolved in the microemulsion exhibits a significantly enhanced ring breathing vibration at ∼815 cm^−^^1^ compared to PIP in pure water, indicating that the microemulsion disrupts the structured hydrogen‐bonding cage that water molecules form around PIP. In pure water, PIP is confined within a tight solvation shell that restricts its conformational and translational freedom. The microemulsion components disrupt this ordered hydration structure, liberating PIP into a less constrained solvation state with greater molecular mobility. The enhanced intensity at ∼880 cm^−^^1^ further confirms this altered solvation environment. This molecular‐level alteration of monomer–solvent interaction is fundamentally impossible when emulsion droplets are merely added to an unmodified aqueous phase, where PIP remains caged within bulk water's hydrogen‐bonding network regardless of what additives are present elsewhere in the system.

UV–vis measurements directly demonstrate the macroscopic consequence of this enhanced molecular freedom on monomer transport (Figure S9). The 1ME system shows notably higher PIP absorption intensity in the 225–230 nm region compared to the control, confirming that more PIP molecules are transported toward and across the reaction interface when dissolved in the microemulsion phase. This enhanced, uniform PIP delivery drives faster polymerization at the interface, leading to rapid film self‐limitation and the entrapment of ∼1 nm microemulsion nanodomains within the polyamide network before they can be expelled which is a critical prior condition for the molecular‐level pore templating mechanism described below.

Surface charge evaluated through zeta potential measurements across a pH range of 3–9 revealed that both control and 1ME‐TFC membranes exhibited negative values throughout the tested range (Figure 1d). The values become more negative at higher pH due to deprotonation of carboxylic acid groups. Notably, the 1ME‐TFC membrane displayed slightly less values compared to the control, particularly at neutral pH (−42 mV vs. −45 mV at pH 7). This difference can be attributed to the trapped microemulsion at the edge of selective layer during the IP process.

Dynamic interfacial tension measurements revealed fundamental differences in how each emulsion system modifies the reaction interface (Figure 1e). The control system maintained almost constant tension at ∼50 mN/m, while coarse emulsion (100ME) showed modest activity, decreasing from 33 to 22 mN/m over 5 min. Nanoemulsion (10ME) shows stronger effects with visible pendant drop deformation. However, microemulsions (1ME) reached ultra‐low interfacial tension (∼0.12 mN/m) within seconds, with captured images showing that the oil drop tends to split and create fluctuating interfaces between solvent and oil, generating bowl‐shaped reaction region as illustrated in Figure 1a. This ultralow tension indicates that 1ME forms a thermodynamically stable single phase rather than maintaining distinct aqueous/organic regions, explaining why microemulsions act as reaction solvents that fundamentally alter polymerization dynamics, while larger emulsions only serve as surface‐modifying additives.

ATR‐FTIR spectroscopy provided additional evidence for the structural modifications induced by solvent effects (Figure S2). All membranes exhibited characteristic polyamide peaks, including the amide I band (C═O stretching) at ∼1660 cm^−^^1^ and amide II band (N─H bending) at ∼1540 cm^−^^1^. Increasing the size of emulsion resulted in a progressive blueshift of the CxO stretching vibration, from 1663 cm^−^^1^ (control) to 1658 cm^−^^1^ (100ME‐TFC). This shift indicates weakening of hydrogen bonding interactions between carbonyl groups and neighboring amide hydrogens, consistent with the hypothesis of the increased free volume within the polymer matrix. The reduced hydrogen bonding density suggests that microemulsion‐templated cavities through active solvent‐mediated IP processes disrupt the typical tight packing of polymer chains, creating a more open network structure.

**FIGURE 2:**
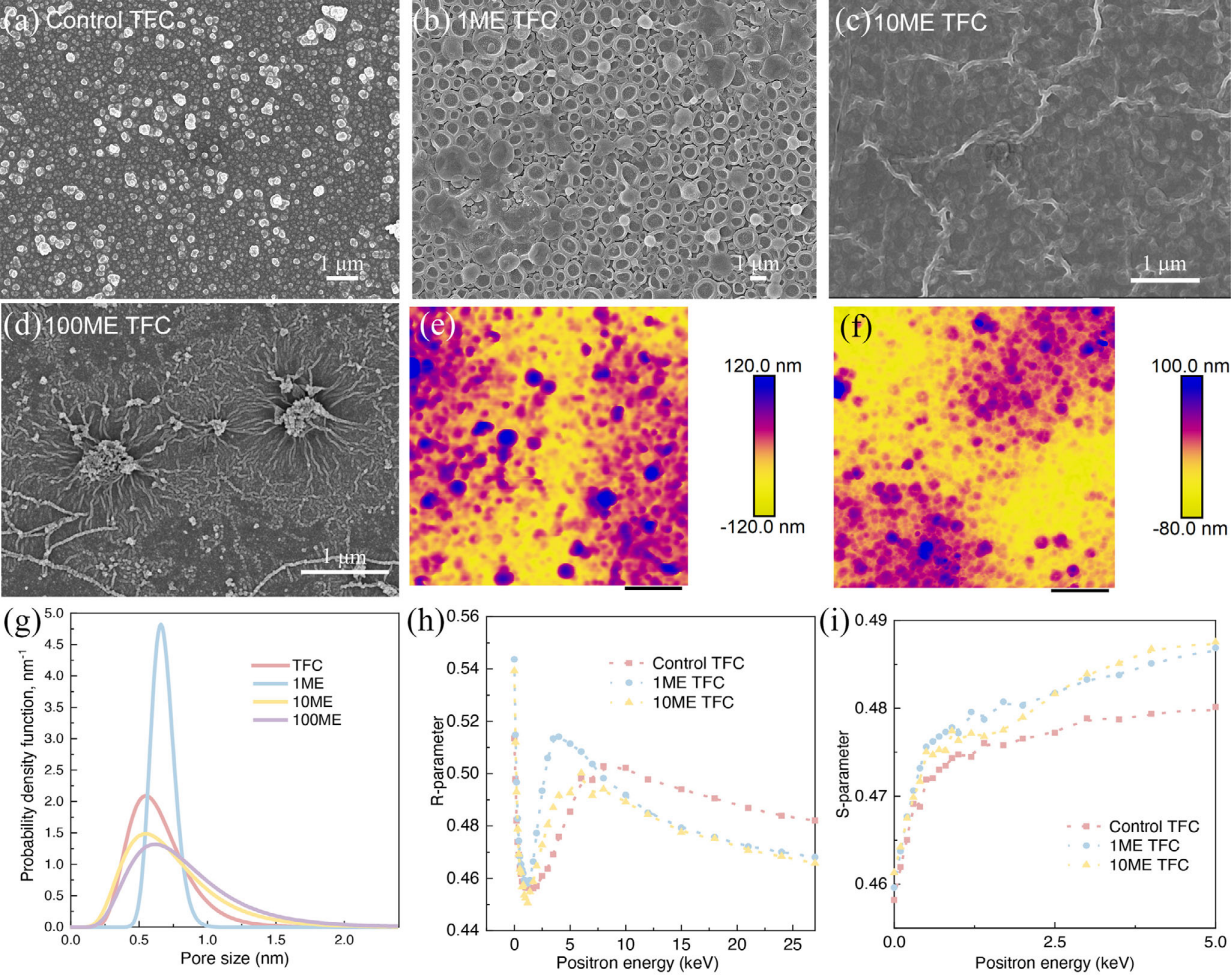
Surface morphological and pore architecture characterization of active solvent‐mediated membrane. (a–d) SEM images showing surface morphology evolution with increasing emulsion sizes: (a) control TFC with nodulus structure, (b) 1ME‐TFC displaying enhanced porosity with bowl‐shaped features, (c) 10ME‐TFC showing pronounced surface cracks and discontinuities, and (d) 100ME‐TFC exhibiting flower‐like crystalline aggregates. Scale bars: 1 µm. AFM height images comparing (e) control TFC and (f) 1ME‐TFC, revealing reduced nanoscale roughness despite increased porosity. Scale bars: 1 µm. (g) Pore size distribution of different membranes. (h) Depth‐dependent R‐parameter profiles demonstrating reduced dense layer thickness and increased void content in active solvent‐mediated membranes. (i) S‐parameter profiles confirming enhanced free volume intensity throughout the membrane depth. Only control, 1ME and 10ME membrane was selected for PAS tests as 100ME membrane only show surface structure changes.

The enhanced hydrophilicity was confirmed by water contact angle measurements (Figure 1f). The control TFC membrane exhibited a contact angle of 62°, typical for polyamide surfaces. Progressive reduction in contact angle was observed with increasing emulsion sizes: 1ME‐TFC (54°), 10ME‐TFC (48°), and 100ME‐TFC (44°). The enhanced hydrophilicity of 10ME‐TFC and 100ME‐TFC can be attributed to the increased surface roughness (in accordance with surface morphologies in Figure 2). These characterization results demonstrate that microemulsion (1ME) as a solvent play different role during IP process comparing to nanoemulsion (10ME) and coarse emulsion (100ME) as additives, potentially changed the surface and pore structure that facilitate water transport [36, 37].

We sought to understand how the active solvent‐mediated process alters the internal pore and surface structure of membrane. The surface morphology of membranes prepared with different emulsion systems were examined using scanning electron microscopy (SEM). As shown in Figure 2a, the control TFC nanofiltration membrane exhibited a typical surface morphology with nodular features. Upon using 1ME as the solvent (Figure 2b), the surface structure underwent significant transformation, developing a more porous appearance with interconnected void networks. The nodular features became less defined, replaced by a bowl‐shaped structure with distinctive surface patterning.

Without the solvent‐mediated effects, increasing the oil droplet size to 10 nm (10ME; nanoemulsion) (Figure 2c) resulted in a dramatically different morphology. The membrane surface exhibited larger‐scale roughness with obvious cracks. When the emulsion size became larger (100ME; coarse emulsion), the surface displayed distinct flower‐like crystalline structures (Figure 2d), indicating that larger oil droplets also changed the polymerization kinetics, suggesting excessive templating agents can lead to uncontrolled polymer aggregation rather than uniform network formation.

Atomic force microscopy (AFM) showed that the control membrane (Figure 2e) has a relatively uniform height distribution across the scanned area across with root‐mean‐square roughness of 36.2 nm (Figure 2e). In contrast, the 1ME‐TFC membrane (Figure 2f) exhibited reduced overall roughness (R_q_ = 26.1) and a narrower height distribution. This reduction in roughness, despite the more porous appearance in SEM, suggests that the active solvent‐mediated interfacial polymerization process creates more uniform nanoscale features while increasing porosity within the polymer matrix.

Pore size distribution analysis revealed the key impact of active solvent‐mediated behavior (Figure 2g). The control TFC membrane exhibited a broad distribution centered at approximately 0.56 nm, characteristic of dense polyamide networks with minimal free volume. Introduction of 1ME significantly narrowed the distribution. This change indicates the successful control of pore architecture upon microemulsion templating. The 10ME membrane showed further peak broadening, while the 100ME membrane displayed the broadest distribution with the peak approaching 0.66 nm. These results demonstrate that only 1ME, or we say microemulsion solvent, could significantly narrow the distribution, indicating successful molecular‐level pore engineering through active solvent‐mediated polymerization.

Positron annihilation spectroscopy (PAS) allowed us to quantify the depth‐dependent microstructural evolution in polyamide layers [38, 39]. The R‐parameter describes the voids density, where a larger R‐parameter means that the voids become larger and/or their quantities increase. The S‐parameter represents the intensity of free volume, where a larger S‐parameter stands for increased free volume cavities and/or a higher free volume. As shown in Figure 2h, the R‐parameters of all membranes decrease dramatically first from ∼0.54, which is usually caused by the back diffusion and scattering of positroniums near the membrane surface. Then, all three membranes exhibit a minimum value before gradually increasing. The Control TFC shows its minimum at 1.7 keV with a value of ∼0.456, while 1ME TFC and 10ME TFC reach lower minima (0.458 and 0.451) at earlier positions (1.2 keV), which means a thinner selective layer. The 1ME solvent‐mediated IP process shows fast recovery of minimum value to plateau, indicating its narrower size distribution, which is in accordance with the results in Figure 2g. In addition, 1ME membrane has the highest average R‐parameter value in the range of surface to end of transition zone, indicating the highest intensity of voids /free volume.

The S‐parameters shown in Figure 2i present a complementary trend. All membranes show an initial rapid increase followed by different plateau behaviors. The Control TFC stabilizes at 0.476 after 1.7 keV before slightly increasing at higher energies. In contrast, the S‐parameters of 1ME TFC and 10ME TFC increase faster with higher values, indicating enhanced free volume in the polyamide selective layer. Considering the S‐parameter corresponding to the positron energy of 0–5 keV usually represents the polyamide layer region [40], we can conclude that 1ME‐TFC with the highest average value of S‐parameter show that 1ME solvent‐mediated IP effectively increased the free volume.

**FIGURE 3:**
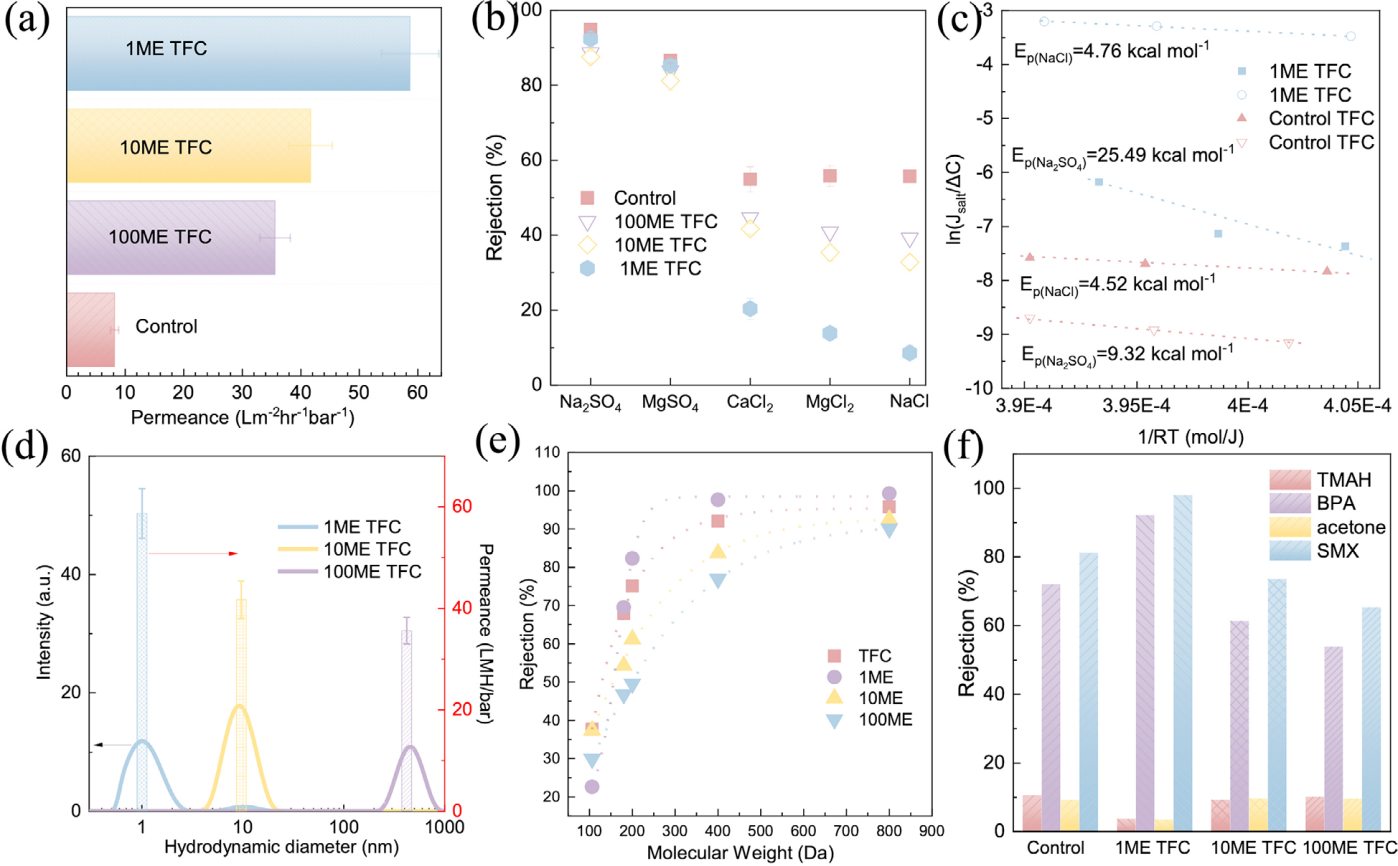
Performance evaluation of active solvent‐mediated nanofiltration membranes. (a) Water permeance and (b) salt rejection performance of different membranes. (c) Arrhenius analysis of salt permeation. (d) Correlation between emulsion size and membrane permeance with size distribution profiles for microemulsion (1ME), nanoemulsion (10ME), and coarse emulsion (100ME). (e) Molecular weight cut‐off tested by neutral solute rejection. (f) Organic compound rejection for simulated semiconductor (TMAH/BPA) and pharmaceutical (SMX/acetone) wastewater treatment, demonstrating tunable selectivity for resource recovery and contaminant removal.

The engineered pore translated directly into superior transport properties. The water permeance of membranes exhibited a strong correlation with emulsion sizes (Figure 3a). The control membrane demonstrated a permeance of 8.2 L m^−^^2^ h^−^^1^ bar^−^^1^. The 1ME‐TFC membrane achieved 58.7 L m^−^^2^ h^−^^1^ bar^−^^1^, representing a 7.1‐fold increase. The performance shows no decline even scaling up to 1‐inch hollow fiber modules (Figure S4), indicating the active solvent‐mediated methods are versatile for both flat sheets and hollow fiber NF membranes fabrication processes. Interestingly, larger emulsion sizes did not show similar effects, with 10ME‐TFC and 100ME‐TFC exhibiting permeances of 41.7 and 35.6 L m^−^^2^ h^−^^1^ bar^−^^1^, respectively. This suggests that only solvent‐mediated effects (with ∼1 nm microemulsions) could significantly change the water permeance, confirming that molecular‐scale templating combined with appropriate solvent domain effects can effectively create water transport channels.

The salt rejection performance (Figure 3b) showed that for divalent anions (Na_2_SO_4_, MgSO_4_), the 1ME‐TFC membrane exhibited slightly reduced but still excellent rejection, indicating that the enhanced free volume did not compromise selectivity for multivalent ions. For monovalent anions (CaCl_2_, MgCl_2_, NaCl), rejection rates were expectedly lower, especially for active solvent‐mediated 1ME‐TFC membrane with NaCl rejection decreased to 8.6%. The selectivity of NaCl/Na_2_SO_4_ for 1ME TFC membrane surpasses state‐of‐the‐art NF membranes, breaking the current upper bound with the highest water permeance and selectivity (Figure S5). Transport behavior analysis through temperature‐dependent permeation studies (Figure 3c) provided further insights into the transport of different ions. Arrhenius plots yielded activation energies of 4.52 and 9.32 kcal mol^−^^1^ for NaCl and Na_2_SO_4_ transport through the control TFC membrane. These values changed to 4.76 and 25.49 kcal mol^−^^1^ through the 1ME TFC membrane. This different behavior is mechanistically consistent with the pore engineering achieved through our active solvent‐mediated approach. The enlarged and interconnected sub‐nanometer pores reduce transport resistance for small monovalent ions (NaCl), while the enhanced Donnan exclusion from the narrower pore size distribution and maintained membrane charge creates a higher energy barrier for divalent SO_4_^2^^−^ ions. This explains how the 1ME‐TFC membrane simultaneously achieves high monovalent salt permeation and excellent divalent salt rejection.

The correlation between emulsion size and membrane performance (Figure 3d) revealed critical structure‐property relationships. Dynamic light scattering measurements confirmed different size distribution of microemulsions (∼1 nm), nanoemulsions (∼10 nm), and coarse emulsions (∼400 nm). The inverse relationship between emulsion size and permeance enhancement suggests that even nanoscale (10 nm) may not effectively modify the polyamide network during interfacial polymerization. Additional performance studies (Figure S3) reveal that higher concentrations of oil (1.5%–3%) in microemulsion show decreased performance, likely due to excessive interface disruption preventing proper polymer network formation or too many interconnected pores which broke the structural integrity. We also applied the active solvent‐mediated methods to fabricate RO membranes. The remarkable performance improvement in RO demonstrates that this kind of strategy benefits extend beyond nanofiltration to tighter membranes (Figure S6). Though the rejection of NaCl decreased from 99.6% to 98.7%, the 3.4‐fold permeance enhancement suggests that the templated pore structure creates more efficient water pathways, suggesting the 1ME RO membrane could be potentially advantageous in brackish water desalination where feed salinity is lower, industrial process water applications with less strict salt requirements, and multi‐pass RO systems where slightly lower rejection per pass is compensated by the significantly higher flux and thus reduced energy consumption. The morphological change is also obvious in RO membranes with smoother surface (Figure S7). All these results indicate that the ∼1 nm microemulsions, not only has small enough size, but also serves as solvent to carry the reactive monomers, creating beneficial solvent domains during polymerization and create molecular‐level pores, while larger oil droplets likely remain at the membrane surface or create defects rather than controlled inner porosity.

Molecular weight cut‐off (MWCO) (Figure 3e) further proved that the pore engineering regulates membrane selectivity. The MWCO and pore size distribution were determined from PEG rejection experiments using a series of molecular weight standards (details could be found in Materials and Methods parts).The control membrane exhibited a sharp rejection curve with MWCO ∼200 Da. The broadening of rejection curves with increasing emulsion sizes indicates not only larger average pore sizes but also wider pore size distributions. However, for 1ME solvent‐mediated IP process, the rejection curve is even sharper than the control one, consistent with the PAS and size distribution results. The 1ME‐TFC membrane maintained relatively sharp size selectivity, suggesting controlled pore formation without excessive defects.

The optimized 1ME membranes with molecular‐level pore engineering show advantages in resource recovery application requiring precise separation. Model compounds representing industrial wastewater was used to test the selectivity of different membranes (Figure 3f). For semiconductor industry simulated waste liquid, tetramethylammonium hydroxide (TMAH, 91 Da) and bisphenol A (BPA, 228 Da; simulation for segments of photoresists) were chosen to simulate semiconductor wastewater, where photoresist fragments foul the resins used for TMAH recovery. The rejection data demonstrated the membrane's potential for TMAH recovery [41, 42]. The control membrane showed 10.2% TMAH and 71.1% BPA rejection, while 1ME‐TFC achieved 3.7% TMAH and 91.2% BPA rejection. The low TMAH rejection is charge and pH driven. TMAH fully dissociates, raising feed pH to ∼11.1 and decreasing the zeta potential. This intensifies attraction of TMA^+^, while the highly mobile co‐ion OH^−^ undergoes facilitated transport. BPA rejection, by contrast, is governed by size exclusion of a neutral molecule. This selective separation capability, combined with 6‐fold higher permeance, suggests significant potential for TMAH recovery from BPA‐containing streams. For pharmaceutical simulated wastewater, sulfamethoxazole (SMX, 253 Da) rejection remained high (>80%) for all membranes, while acetone (58 Da) rejection varied from 12.4% (control) to 3.2% for 1ME membrane. This selectivity profile enables efficient antibiotic removal while allowing solvent passage, beneficial for pharmaceutical waste treatment and resource recovery during manufacturing processes.

The active solvent‐mediated influence on membrane structure extends beyond simple physical templating, as directly demonstrated by the Raman and UV–vis evidence above. The microemulsion does not only carry PIP passively, it fundamentally alters PIP's solvation environment, enhances its transport to the interface, and accelerates polymerization. This accelerated, uniform film formation entraps the ∼1 nm microemulsion nanodomains within the polyamide matrix, creating the molecular‐level pore templating that we now characterize computationally. This effect along with the creation of organized solvent domains, explains the dramatic enhancement in membrane performance.

**FIGURE 4:**
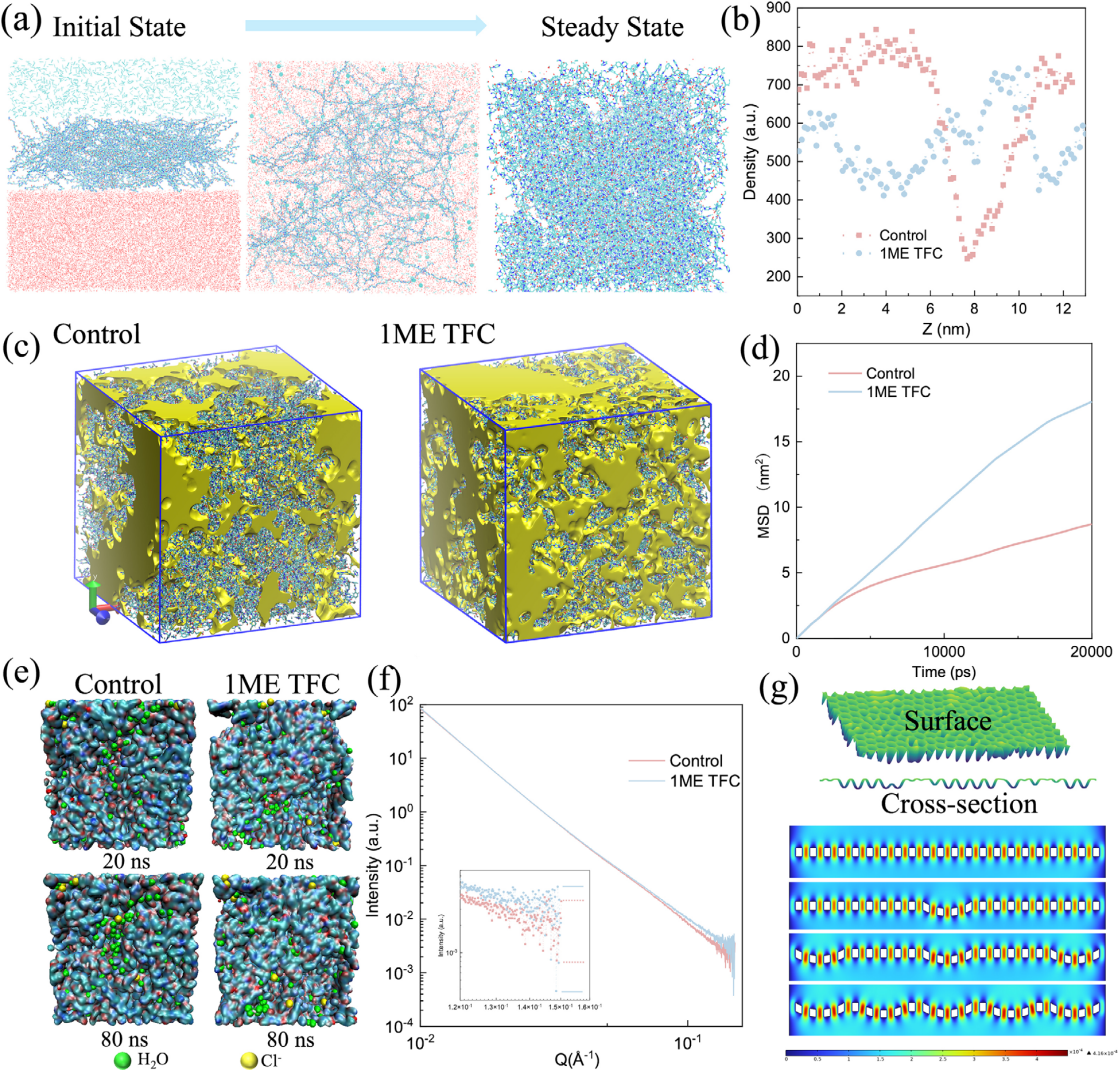
Molecular dynamics simulations and multi‐scale structural analysis of membrane formed via active solvent‐mediated interfacial polymerization. (a) MD simulation showing polymer network formation from initial random distribution to equilibrated steady‐state structure. (b) Density profiles of polymers along the z‐axis. (c) Free volume evolution demonstrating 11.2% enhancement in 1ME‐TFC membrane vs. control. The yellow parts represent calculated free volume. (d) Mean square displacement of Cl^−^ molecules in 1ME‐TFC membrane and control membrane. (e) schematic illustration of water and Cl^−^ transport pathways through the polyamide matrix, showing how the interconnected free volume channels in the 1ME‐TFC system provide preferential pathways for molecular transport. (f) SAXS profiles of control and 1ME‐TFC membranes. (g) CFD simulations revealing concentrated flow channels within bowl‐shaped structures that enhance water permeance.

Molecular dynamics simulations provided molecular‐level insights into the structural changes. The simulation protocol (Figure 4a) initiated with randomly distributed polymer chains in a periodic box, which underwent energy minimization and equilibration to reach steady‐state configurations. The transition from the initial dispersed state to the final cross‐linked network clearly demonstrates the formation of polyamide selective layer. Density profile analysis along the z‐axis (Figure 4b) revealed pore architecture control mediated by the microemulsion solvent. The 1ME‐TFC membrane exhibited a general narrow and uniform polymer density distribution ranges from 411–741 along the z‐axis. In contrast, the control membrane showed an obvious decrease of density along the z‐axis 6–11 nm region, indicating the presence of significant density fluctuations, which is consistent with the broader size distribution.

Free volume calculation results (Figure 4c) provided quantitative data showing the 1ME‐TFC membrane achieved a free volume of 11.2% increase compared to the control, equaling to ∼1.6 times enhancement in water permeance. This increasement could not fully explain the tested 7.1 times enhancement in total water permeance, suggesting there are other reasons, for example increased pore connection, contributes to the overall performance enhancement. Mean square displacement (MSD) calculations further revealed the differences in transport behavior (Figure 4d). Cl^−^ ions in the 1ME TFC membrane exhibited significantly enhanced ion mobility, with MSD reaching approximately 18 nm^2^ after 20 ns. In contrast, the control Membrane showed restricted motion, reaching only about 8 nm^2^ over the same time. The difference in ion mobility suggests that the active solvent‐mediated process generates channels with less resistance and more efficient pathways for water and monovalent ions transport, contributing to the higher permeance as observed in the experiment. Visual representations of molecular trajectories illustrated these mechanistic differences (Figure 4e). After 80 ns simulation, the control membrane showed less Cl^−^ ions left in the simulation box, consistent with the slower ion transport observed in the MSD plot. However, the 1ME TFC membrane showed more Cl^−^ ions penetrating through the simulation box, confirming the faster transport of smaller ions through the selective layer.

We tried to experimentally verify these computational results using small‐angle x‐ray scattering (SAXS) to probe the pore structure of polyamide selective layers. As shown in Figure 4f, both the Control and 1ME TFC membranes exhibit similar scattering behaviors in the low Q region. However, a distinct difference is observed in the high Q region (0.13–0.15 Å^−^^1^), where the 1ME TFC membrane displays a higher scattering intensity and broadening of signal compared to the control. This difference indicates a higher density of pores and increased polydispersity within the 1ME TFC polymer matrix [43, 44], which directly confirm the pore engineering successfully created more interconnected pores. Taken together with the above result, we now get the validated mechanism of molecular‐level pore engineering induced by active solvent. The microemulsion liberates PIP from its hydrogen‐bonding hydration cage, enhancing uniform monomer delivery to the reaction interface, which drives rapid polymerization and early self‐limitation producing a thinner selective layer, entraps the ∼1 nm nanodomains as structural templates, and forms an interconnected pore network causing the observed permeance enhancement.

To further bridge molecular‐level insights with macroscopic performance, we employed computational fluid dynamics (CFD) simulations to explore the hydrodynamic changes of active solvent‐mediated surface structures (Figure 4g). Surface morphologies were generated by VisualPDE [45] using the Gray‐Scott reaction‐diffusion model, with monomer diffusion coefficients (*D_u_* and *D_v_*, explained in *Materials and Methods*) adjusted to reflect the active solvent‐mediated interfacial polymerization process based on our hypothesis. Interestingly, the visualized results (Figure 4g upper) revealed bowl‐shaped structure in the 1ME‐TFC membrane, perfectly match cross‐section image of 1ME selective layer (Figure S1). Flow field simulations through these structures demonstrated that bowl‐shaped depressions create localized flow enhancement zones. Color‐coded velocity profiles showed concentrated flow channels within the idealized water channels, with velocities up to threefold higher than in surrounding regions. The control membrane, lacking these depressions, exhibited uniform vertical flow through pores. Introducing a single depression generated ∼3% (Table S1) localized flow enhancement, where water molecules channeled into the modified region before redistributing. As depression density increased to five per simulation domain, interconnected high‐flux zones developed with maximum velocities approaching 11% enhancement. These CFD results give a qualitative result demonstrating how active solvent‐mediated structures effectively increase the permeability in a macroscopic level.

## 3 Conclusion

Active solvent‐mediated interfacial polymerization creates molecular‐level modifications in polyamide membranes through physical templating and modulation of local reaction environments. The success of this approach highlights the critical importance of understanding and exploiting solvent effects in interfacial polymerization. Beyond serving as simple templates, the microemulsion components create complex solvent environments that actively participate in directing polymerization pathways. Comprehensive characterization validates the creation of enhanced free volume (11.2% increase), increased interconnected pore densities, narrowed pore size distribution. These structural modifications translate directly to 7.1‐fold enhancement in water permeance while modulating selectivity, with demonstrated ability in TMAH recovery and pharmaceutical/small organics separation. This approach opens new avenues for membrane design where selection of microemulsion components based on their solvent properties can provide control over membrane structure and performance. By pore engineering at the molecular level through combined templating and solvent effects, membranes can be designed to meet increasingly diverse demands for water treatment and industrial separations.

## Author Contributions

F.L. conceived the idea of the research. F.L. and DY.H designed the experiments. F.L. contributed to the synthesis of microemulsions and the membranes. F.L. carried out the batch experiments and characterization. YX.M and F.L. conducted the MD and CFD simulations. F.L., DY.H., RMGR, and M.Y contributed to the manuscript writing and discussions.

## Conflicts of Interest

The authors declare no conflicts of interest.

## Supporting information




**Supporting File**: advs77014‐sup‐0001‐SuppMat.docx.

## Data Availability

The data that support the findings of this study are available from the corresponding author upon reasonable request.
