# Supplementary material for: Molecular‐Level Pore Engineering for Precise Separation via Active Solvent‐Mediated Interfacial Polymerization
Source: Adv Sci (Weinh). 2026 Aug 5;13(60):e77014. doi: 10.1002/advs.77014 (PMC13440197; doi:10.1002/advs.77014)
Supplement: Supplementary file 1 [file ADVS-13-e77014-s001.docx]

**Supporting Information**

**Molecular-level pore engineering for precise separation via active solvent-mediated interfacial polymerization**

Feng Li^[a,b]^, R.M.G. Rajapakse^[c]^, Yuxin Mou^[a]^, Deyin Hou* ^[a,b]^ and Min Yang^[a,b]^

*^[a]^National Engineering Research Center of Industrial Wastewater Detoxication and Resource Recovery, Research Center for Eco-Environmental Sciences, Chinese Academy of Sciences, Beijing 100085, PR China.*

*^[b]^University of Chinese Academy of Sciences, 100049, Beijing, China*

*^[c]^* *Department of Chemistry, University of Peradeniya, Peradeniya 20400, Sri Lanka*

*Corresponding authors: Professor Deyin Hou [dyhou@rcees.ac.cn](mailto:dyhou@rcees.ac.cn)

The supplementary information contains Materials and Methods part, 1 table and 7 figures.

**Materials and Methods**

**Materials**

Polyethersulfone (PES) ultrafiltration membranes (MWCO: 20 kDa, RisingSun Membrane Technology, China) were used as substrate supports. Trimesoyl chloride (TMC, >98%, TCI Chemicals, Japan), piperazine (PIP, >99%, Aladdin, China), and n-hexane (>99.5%, Sinopharm Chemical Reagent, China) were purchased at the highest available purity. For microemulsion preparation, sodium dodecyl sulfate (SDS, >99%, Sigma-Aldrich), n-butanol (>99.5%, Aladdin) were used as received. Salts for performance testing including NaCl, Na₂SO₄, MgCl₂, and MgSO₄ (Sinopharm) were of analytical grade. Tetramethylammonium hydroxide (Sinopharm), bisphenol A (BPA, Aladdin), and sulfamethoxazole (>98%, Sigma-Aldrich) were used for separation studies. All chemicals were used without further purification. Ultrapure water (18.2 MΩ·cm) was obtained from a Milli-Q system (Millipore).

**Preparation of Microemulsions**

Microemulsions were prepared following an oil-in-water (O/W) system using SDS as surfactant, 1-butanol as co-surfactant, n-hexane as the oil phase, and deionized water. The microemulsion composition was optimized to achieve thermodynamically stable systems with droplet sizes of ~1 nm. For the 1ME system, the weight ratio was SDS:1-butanol:n-hexane:water = 8:8:1:83. Dynamic light scattering (DLS) measurements confirmed microemulsion droplet sizes of 1.0 ± 0.2 nm for the optimized formulation. Thermodynamic stability was verified by monitoring the microemulsions for phase separation over 30 days. For comparison, larger emulsions (10ME, ~10 nm, 100ME, ~400 nm) were prepared by adjusting oil concentration and using high-pressure homogenization.

**Preparation of TFC Membranes**

TFC membranes were fabricated via interfacial polymerization with or without different emulsion systems. The PES support was fixed in a custom-designed frame with an effective area of 4 × 4 cm². For control membranes, an aqueous solution containing 0.5 wt% PIP was poured onto the support and allowed to soak for 2 minutes. Excess solution was removed. Subsequently, 0.15 wt% TMC in n-hexane was gently poured onto the membrane surface for 1 minute to enable interfacial polymerization. For active solvent-mediated membranes (ME-TFC), the aqueous PIP solution was replaced with microemulsion containing equivalent PIP concentration. Different hexane loadings (0.5, 1.0, 1.5 and 3.0 wt%) were investigated. For aqueous PIP containing nanoemulsion (10ME) and coarse emulsion (100ME), the procedure was similar to the preparation of control TFC membrane. The hollow fiber control and 1ME TFC membranes were fabricated using the same materials and reaction procedure as the flat sheet membranes. Detailed operation procedure for hollow fiber membranes can be found in our previous work^[1]^. After polymerization, all membranes were washed with n-hexane to remove unreacted TMC, followed by thorough rinsing with ultrapure water. The membranes were then heat cured at 60°C for 5 minutes to enhance crosslinking and stored in ultrapure water at 4°C until characterization.

**Membrane Performance Evaluation**

Membrane performance was evaluated using a crossflow filtration system with four parallel test cells. Tests were conducted at 25 ± 0.5°C with a crossflow velocity of 0.5 L/min and transmembrane pressure of 5 bar. Feed solutions contained various salts, organics or organics/salts mixtures . Prior to data collection, membranes were pre-compacted at 6 bar for 60 minutes to ensure steady operation. Water permeance ($J)$was calculated using:
$J (Lm^{-2}h^{-1}\mathrm{bar}^{-1})=\frac{\Delta V_{p}}{(A_{m}\cdot\Delta t\cdot\Delta P)}$	(1)

where $\Delta V_{p}$ (L) is change in permeate volume collected over the time interval $\Delta t$ (h), $\Delta P$ (bar) is the transmembrane pressure, $A_{m}$ (m^2^) is the active membrane areas.

Salt rejection (R) was determined from:
$R\left( \% \right)=\left( 1-\frac{C_{p}}{C_{f}} \right)\times100\%$	(2)

where ($C_{f})$ and ${(C}_{p})$are salt concentrations in feed and permeate, respectively, measured by conductivity. Rejection of organic contaminants was evaluated using 100 ppm solutions of TMAH, BPA, and SMX. TMAH rejection was determined using ion chromatography (Dionex ICS-5000+) equipped with a conductivity detector and IonPac CS17 analytical column. BPA and SMX rejection were measured by high-performance liquid chromatography (HPLC, Agilent Series 1100) with ZORBAX Original C18 reverse-phase column.

**Characterization**

Surface and cross-section morphologies were examined by field emission scanning electron microscopy (FESEM, Hitachi SU8010) after platinum coating. Surface topology was characterized by atomic force microscopy (AFM, Bruker Multimode -HR8) in tapping mode over 5 × 5 μm² areas. Surface roughness parameters (R_a_, R_q_) were calculated using NanoScope Analysis software. Surface chemistry was analyzed by X-ray photoelectron spectroscopy (XPS, ThermoFisher ESCALAB 250Xi). Attenuated total reflection Fourier transform infrared spectroscopy (ATR-FTIR, Bruker INVENIO S) was performed from 4000-600 cm⁻¹ at 4 cm⁻¹ resolution. Zeta potential of membrane surfaces was measured using Anton Paar Surpass 3. The pore size distribution of the nanofiltration membranes was calculated using neutral solute rejection results, with details shown in Supplementary Text II. Doppler broadening energy spectroscopy (DBES) with a variable mono-energy slow positron beam ^22^Na source) was used to probe the microstructural evolution across the membrane cross-section. The depth profile was established by collecting 30 spectra across an incident energy sweep from 0 to 27 keV. The resulting S- and R-parameters were extracted to quantify changes in free volume and pore size distribution as a function of positron penetration depth. SAXS profiles were acquired on a Xenocs Xeuss Pro beamline using Cu Kα radiation (λ = 1.54 Å). The scattered X-rays were detected by a 2D detector. Data reduction, including background subtraction and azimuthal integration, was performed using the XSACT analysis software.

**Dynamic interfacial tension**

Dynamic interfacial tension measurements were performed to investigate the influence of emulsion systems on interfacial polymerization kinetics. Pendant drop tensiometer was used for moderate tensions (>1 mN/m) and spinning drop tensiometer was used for ultra-low tensions (<1 mN/m). For control, nanoemulsion (10ME), and coarse emulsion (100ME) systems, a pendant drop tensiometer (Kino SL200) was utilized. Oil droplets (hexane) containing TMC were formed in PIP/emulsion solution. For microemulsion (1ME) systems exhibiting ultra-low interfacial tensions, a spinning drop tensiometer was used (Kino TX500). A small droplet of hexane with TMC was injected into a capillary tube filled with microemulsion solvent. The tube was rotated at 6000-8000 rpm, with rotation speed optimized to achieve a droplet length-to-diameter ratio between 4:1 and 8:1. For both methods, baseline measurements were first established with emulsion-free systems, followed by continuous monitoring during testing.

**Pore size distribution of different membranes**

Membrane pore size distributions were characterized through neutral solute rejection experiments using polyethylene glycol (PEG) molecular weight standards. Feed solutions containing 50 mg/L PEG were filtered at 1.0 bar transmembrane pressure, 25.0 ± 0.5°C, and 20.0 cm/s crossflow velocity. The effective solute diameter (ds) was determined from molecular weight (M) according to:

$$log\frac{d_{s}}{2}=-1.4962+0.4654logM (S1)$$

where M is in Da and ds in meters. Rejection coefficients were calculated from total organic carbon measurements of feed and permeate samples.

The pore size distribution followed a log-normal probability density function. The mean effective pore size (μp) corresponded to the solute diameter at 50% rejection, while the geometric standard deviation (σp) was obtained from the ratio of solute diameters at 84.13% and 50% rejection. The MWCO was defined as the molecular weight corresponding to 90% solute rejection. The distribution function is given by:

$$\frac{dR(d_{p})}{{dd}_{p}}=\frac{1}{d_{p}In\sigma_{p}\sqrt{2\pi}}\exp\left[ -\frac{(Ind_{p}-In\mu_{p})^{2}}{2(In\sigma_{p})^{2}} \right] (S2)$$

**Molecular Dynamics Simulations**

MD simulations were performed using GROMACS version 2022.5^[2]^ with the GAFF force field to investigate microemulsion effects on polyamide membrane structure. Trajectory visualization and analysis were conducted using VMD version 1.9.4a57^[3]^. Three systems were modeled, control polyamide with 120 polymer chains (10 repeating units each), polyamide with 100 model microemulsions, and polyamide with 500 model microemulsions (equal to ~1% microemulsion). All systems contained n-hexane (organic phase) and water molecules (~170,000-200,000 total atoms). Systems were equilibrated for 60 ns (NPT ensemble) followed by 20 ns free volume analysis (NVT ensemble). Subsequently, 2,000 ppm NaCl solution was introduced for 80 ns production runs to evaluate ion transport. Diffusion coefficients for water and Cl⁻ ions, along with free volume distributions, were calculated from production trajectories. Molecular topologies were generated using AuToFF software^[4]^, with partial charges calculated via the AM1-BCC method. Force field parameters were assigned using the General AMBER Force Field (GAFF) ^[5]^. More details could be found in the Supplementary Text III.

**Computational Fluid Dynamics Simulations**

Fluid-Structure Interaction (FSI) numerical simulations were performed using COMSOL Multiphysics to investigate water permeation through nanofiltration membrane surfaces. Representative 2D computational units were constructed based on typical nanofiltration membrane surface characteristics to model localized flow processes. Given the quasi-periodic distribution of membrane surface structure at macroscale, a periodic simplification strategy was employed to balance computational accuracy and efficiency. Representative units containing multiple consecutive structures were selected. Surface depression structures were varied (0, 1, 3, and 5 depressions) to investigate their impact while maintaining model physical fidelity and computational feasibility.

Fluid phase behavior was modeled as incompressible Newtonian flow, governed by the continuity equation and Navier-Stokes equations:

$$\begin{aligned} \nabla\cdot u=0\#(S3) \end{aligned}$$

$$\begin{aligned} \rho\left( u\cdot\nabla\right)u=-\nabla p+\mu\nabla^{2}u\#(S4) \end{aligned}$$

where **u** is the velocity vector, p is pressure, ρ is water density, and μ is dynamic viscosity. Water properties were assumed constant under simulation conditions, excluding temperature variations and solute transport to isolate pure water permeation behavior at pore scale.

The solid phase employed polyamide material properties, with mechanical parameters (Young's modulus and Poisson's ratio) assigned based on literature-reported values for thin-film composite membranes.

Velocity inlet boundary conditions were applied to simulate transmembrane pressure-driven permeation during nanofiltration operation. The outlet was specified as a pressure outlet boundary. No-slip conditions were imposed on all pore walls and channel surfaces, assuming zero fluid velocity at solid interfaces.

Steady-state simulations were performed with both fluid and solid domains discretized using unstructured meshes. Local mesh refinement was applied within surface and near pore walls to accurately capture high-velocity permeation features and pressure gradient distributions.

Prior to production simulations, mesh independence analysis was conducted by systematically refining mesh density (normal, fine, extra fine) as shown in Table S1. Maximum pore velocity (Umax) was monitored as the primary convergence criterion. When parameter variations with further refinement fell below the predetermined threshold (≤1%), the solution was considered mesh-independent, establishing the fine mesh configuration for subsequent calculations.

**Table S1** Mesh sensitivity results for the max velocity inside the membrane pore.

| Model | Mesh level | Cells | $U_{max}$ (m/s) | Variation (%) |
| --- | --- | --- | --- | --- |
| Model 1 | Coarse | 15548 | 3.75*10^-4^ | ≤1% |
| Model 1 | Fine | 23174 | 3.75*10^-4^ | ≤1% |
| Model 1 | Extra-fine | 73376 | 3.75*10^-4^ | ≤1% |
| Model 2 | Coarse | 15502 | 3.89*10^-4^ | ≤1% |
| Model 2 | Fine | 23144 | 3.89*10^-4^ | ≤1% |
| Model 2 | Extra-fine | 73486 | 3.89*10^-4^ | ≤1% |
| Model 3 | Coarse | 15522 | 4.09*10^-4^ | ≤1% |
| Model 3 | Fine | 23166 | 4.09*10^-4^ | ≤1% |
| Model 3 | Extra-fine | 73448 | 4.09*10^-4^ | ≤1% |
| Model 4 | Coarse | 15366 | 4.16*10^-4^ | ≤1% |
| Model 4 | Fine | 23414 | 4.16*10^-4^ | ≤1% |
| Model 4 | Extra-fine | 73298 | 4.16*10^-4^ | ≤1% |

**Gray-Scott reaction-diffusion model**

To elucidate the role of monomers in modulating interfacial polymerization dynamics, we employed the Gray-Scott reaction-diffusion model:

$\frac{\partial u}{\partial t}= D_{u}\nabla^{2}u-uv^{2}+F(1-u)$	(S5)

$\frac{\partial v}{\partial t}= D_{v}\nabla^{2}v+uv^{2}-(F+k)v$	(S6)

where $u$ and $v$represent the concentrations of two chemical species, varying with time $t$ and spatial position $x$. $D_{u}$ and $D_{v}$are the diffusion coefficients of the two species. *F* is the feed rate, representing the rate at which species $u$ is supplied to the system. *k* is the kill rate, representing the rate at which species $v$ is removed from the system. $\nabla^{2}$ is the Laplace operator, characterizing diffusion in space. Within the microemulsion framework, the key parameters *F* and *k* are significantly influenced compared to conventional interfacial polymerization. The active solvent-mediated process effectively modulates F by creating localized high-concentration zones of monomers, while simultaneously affecting *k* through enhanced reaction kinetics within the confined nanoscale domains.

**Fig.S1.** **Cross-section morphology of different membranes.** (a) 1ME-TFC membrane exhibiting bowl-shaped structure within the selective layer, with interconnected voids throughout the polyamide matrix. (b) Control TFC membrane showing typical dense polyamide layer with nodulus-like surface morphology. Scale bars: 1 μm.

**Fig. S2.** FTIR spectra of membranes prepared with emulsion containing various oil concentrations. It should be noted that 1ME represents the microemulsion with droplet size ~1 nm, 10ME represents nanoemulsion with droplet size ~10 nm, 100ME represents coarse emulsion with droplet size >100 nm.

**Fig. S3. Optimization of microemulsion concentration for membrane performance.** (a) Water permeance comparison across different 1ME concentrations (0.5%, 1%, 1.5%, 3% v/v). (b) Salt rejection performance for various salts (Na₂SO₄, MgSO₄, CaCl₂, MgCl₂, NaCl) as a function of 1ME concentration.

Performance studies reveal an optimal microemulsion concentration. The 1% 1ME formulation achieves maximum water permeance while maintaining excellent selectivity. Higher concentrations (1.5-3%) show decreased performance, likely due to excessive interface disruption preventing proper polymer network formation or too many interconnected pores which broke the structure integrity.

**Fig. S4.** Scalable fabrication of 1ME hollow fiber membranes. (a) Laboratory-scale hollow fiber modules. (b) Performance test using two 1-inch 1ME hollow fiber modules.

The successful scaling up to 1-inch hollow fiber NF modules demonstrates the industrial viability of active solvent-mediated interfacial polymerization. The 1-inch modules maintain consistent performance across multiple fabrication batches, confirming process reproducibility.

**Fig. S5.** Performance comparison with state-of-the-art membranes. Upper bound plot for NaCl-Na_2_SO_4_ selectivity versus water permeance, comparing 1ME-TFC membrane with literature data for polyamide membranes ^[6]^. The dashed red line indicates the current upper bound limit.

**Fig. S6.** Performance of 1ME solvent-mediated RO membranes. Water permeance and NaCl rejection comparison between control and 1ME-TFC RO membranes tested at 10 bar with 2000 ppm NaCl solution at 25°C. The 1ME-TFC RO membrane achieves 4.1 Lm⁻²h⁻¹bar⁻¹ water permeance while maintaining 98.1% NaCl rejection, representing a 3.4-fold permeability enhancement over the control (1.2 Lm⁻²h⁻¹bar⁻¹, 98.9% rejection).

The remarkable performance improvement in RO demonstrates that this kind of active solvent-mediated process benefits extend beyond nanofiltration to tighter membranes. The high salt rejection with 3.4-fold permeance enhancement suggests that the templated pore structure creates more efficient water pathways without compromising selectivity.

**Fig. S7.** Surface morphology comparison of RO membranes. SEM images showing (a) control RO membrane with typical ridge-and-valley structure characterized by sharp, well-defined features, and (b) 1ME-TFC-RO membrane displaying smoother, more interconnected surface topology with reduced ridge height and broader valley regions. Scale bars: 1 μm.

The morphology change induced by 1ME solvent-mediated is also obvious in RO membranes. The control exhibits the characteristic ridge-and-valley structure. In contrast, the 1ME-RO surface shows a different architecture with smoother surface. This morphology suggests that microemulsions not only template the internal pore structure but also modify the surface roughness, potentially reducing fouling propensity while enhancing water transport.

**Figure S8** Raman shift of PIP in pure water and microemulsion.

**Figure S9** UV-vis spectrum of PIP in control and 1ME TFC system.

References:

[1] F. Li, C. Fang, W. Liu, L. Yang, B. Guo, S. Zhang, High recovery, energy efficient wastewater desalination. *Journal of Membrane Science* **2021**, *631*, 119317.

[2] M. J. Abraham, T. Murtola, R. Schulz, S. Páll, J. C. Smith, B. Hess, E. Lindahl, GROMACS: High performance molecular simulations through multi-level parallelism from laptops to supercomputers. *SoftwareX* **2015**, *1*, 19-25.

[3] W. Humphrey, A. Dalke, K. Schulten, VMD: visual molecular dynamics. *Journal of Molecular Graphics* **1996**, *14*, 33-38.

[4] C. Wang, K. Liao, Z. Wang, Y. Wang, K. Gong, AuToFF Program, Version 1.0. Hzwtech. Shanghai **2023**. Software available at: https://cloud.hzwtech.com/web/product-service?id=36.

[5] J. Wang, R. M. Wolf, J. W. Caldwell, P. A. Kollman, D. A. Case, Development and testing of a general amber force field. *Journal of Computational Chemistry* **2004**, *25*, 1157-1174.

[6] Z. Yang, L. Long, C. Wu, C. Y. Tang, High permeance or high selectivity? Optimization of system-scale nanofiltration performance constrained by the upper bound. *ACS ES&T Engineering* **2021**, *2*, 377-390.
